# Reactive centre loop mutants of α-1-antitrypsin reveal position-specific effects on intermediate formation along the polymerization pathway

**DOI:** 10.1042/BSR20130038

**Published:** 2013-06-25

**Authors:** Imran Haq, James A. Irving, Sarah V. Faull, Jennifer A. Dickens, Adriana Ordóñez, Didier Belorgey, Bibek Gooptu, David A. Lomas

**Affiliations:** *Department of Medicine, University of Cambridge, Cambridge Institute for Medical Research, Wellcome Trust/MRC Building, Hills Road, Cambridge CB2 0XY, U.K.; †Institute of Structural and Molecular Biology, Birkbeck, University of London, London, U.K.

**Keywords:** cirrhosis, emphysema, FRET, intermediate, polymerization, serpin, ANS, 8-anilinonaphthalene-1-sulfonic acid, bis-ANS, 4,4′-dianilino-1,1′-binaphthyl-5,5′-disulfonic acid, FRET, Förster resonance energy transfer, NTA, nitrilotriacetic acid, RCL, reactive centre loop, SI, stoichiometry of inhibition, T_m_,midpoint of thermal denaturation

## Abstract

The common severe Z mutation (E342K) of α_1_-antitrypsin forms intracellular polymers that are associated with liver cirrhosis. The native fold of this protein is well-established and models have been proposed from crystallographic and biophysical data for the stable inter-molecular configuration that terminates the polymerization pathway. Despite these molecular ‘snapshots’, the details of the transition between monomer and polymer remain only partially understood. We surveyed the RCL (reactive centre loop) of α_1_-antitrypsin to identify sites important for progression, through intermediate states, to polymer. Mutations at P_14_P_12_ and P_4_, but not P_10_P_8_ or P_2_P_1′_, resulted in a decrease in detectable polymer in a cell model that recapitulates the intracellular polymerization of the Z variant, consistent with polymerization from a near-native conformation. We have developed a FRET (Förster resonance energy transfer)-based assay to monitor polymerization in small sample volumes. An *in vitro* assessment revealed the position-specific effects on the unimolecular and multimolecular phases of polymerization: the P_14_P_12_ region self-inserts early during activation, while the interaction between P_6_P_4_ and β-sheet A presents a kinetic barrier late in the polymerization pathway. Correspondingly, mutations at P_6_P_4_, but not P_14_P_12_, yield an increase in the overall apparent activation energy of association from ~360 to 550 kJ mol^−1^.

## INTRODUCTION

**Figure 1:**
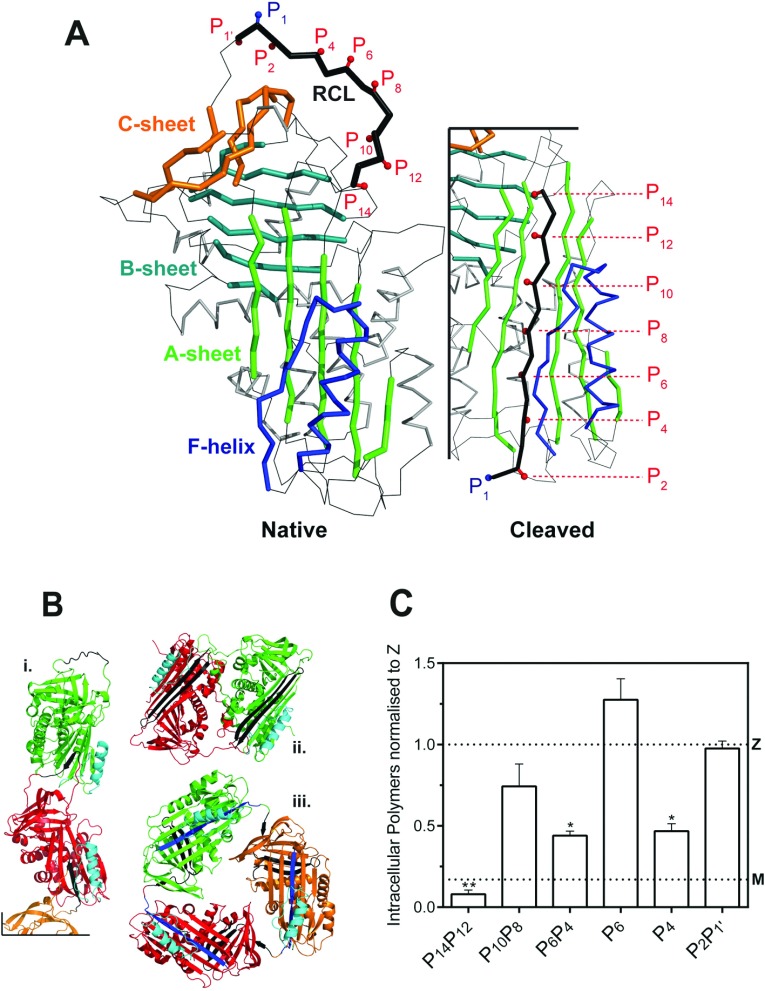
RCL mutants of α_1_-antitrypsin (**A**) The location of the mutations used in this study are indicated against a cartoon representation of wild-type α_1_-antitrypsin (prepared using PyMol and PDB entries 1QLP [42] and 1EZX [43]). The RCL is highlighted in black, and numbered according to the P-site convention of Schechter and Berger [28], in which the P_1_ and P_1′_ positions are either side of the site of cleavage by a cognate protease. The three β-sheets of the serpin fold and the F-helix are labelled. Upon transition from the native, active conformation (left panel) to the cleaved or latent forms, the RCL moves from an exposed to an inserted position as an additional β-strand of β-sheet A (right panel). (**B**) The three main models of polymerization: i, a molecular model of a loop-sheet polymer; ii, the crystal structure of a closed dimer of antithrombin; iii, a closed trimer of α_1_-antitrypsin. The site of inter-molecular interaction in each form is shown in black and helix F is coloured cyan. (**C**) Mutations at positions P_14_P_12,_ P_6_P_4_ and P_4_ of the RCL reduce intracellular polymerization of Z α_1_-antitrypsin. COS-7 cells were transiently transfected with M α_1_-antitrypsin, Z α_1_-antitrypsin and RCL mutants on a Z α_1_-antitrypsin background in serum-free medium for 24 h before lysis. A sandwich ELISA analysis of intracellular polymers made use of the polymer-specific 2C1 monoclonal antibody. The results are normalized to Z α_1_-antitrypsin (*n*=4; mean±S.D.) and differences were assessed by one-way ANOVA with Bonferroni's correction for multiple comparisons: *, *P*<0.01; **, *P*<0.001. The value for M α_1_-antitrypsin is shown with a dotted line (M).

The serine protease inhibitor (serpin) superfamily plays important roles in controlling a wide range of proteolytic cascades [1]. A serpin in its inhibitory form can be viewed as a metastable folding intermediate that converts to an extremely stable state on proteolytic cleavage of an exposed ‘RCL’ (reactive centre loop), as summarized in Figure 1(A). This transition forms the basis of the serpin inhibitory mechanism [2]. Point mutations that perturb the balance between metastable and stable states result in diseases, termed serpinopathies, in which ordered polymers are retained within the endoplasmic reticulum of the cell of synthesis [3], and aggregate as inclusions associated with toxicity and cell death [4]. The archetypal serpinopathy is α_1_-antitrypsin deficiency, in which the affected individuals can develop neonatal hepatitis and cirrhosis and early onset emphysema [5].

Polymers can be induced *in vitro* at elevated temperatures [6,7], by proteolytic digestion following the P_6_, P_7_ or P_10_ residues [8–10], by low concentrations of denaturant [6,7], by a peptide mimetic of the P_14_–P_9_ residues of the RCL [11,12] or at low pH [13]. A mechanism has been proposed, which describes the transition from monomer (*M*) to polymer (*P*) in which the monomer is ‘activated’ to an intermediate state (*I*_pol_), which then self-associates (*P*) or is converted into a monomeric inactive form (*L*) [14–16]:


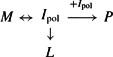


During the activation to intermediate, there is a change in far-UV circular dichroic signal, intrinsic tryptophan fluorescence, the interaction with ANS (8-anilinonaphthalene-1-sulfonic acid) or bis-ANS (4,4′-dianilino-1,1′-binaphthyl-5,5′-disulfonic acid) dyes [14,17], ion mobility mass spectrum collision cross-sectional area [18] and NMR cross-peaks [19]. While denaturant can induce polymerization at concentrations that favour population of an unfolding intermediate state (*I*_denat_) [6,20], observations indicate that there are multiple pathways favoured depending on the manner in which polymers are formed, and as a result intermediate ensembles represented by *I*_pol_ and *I*_denat_ could be structurally distinct [19]. In support of this, it has recently been shown that polymers produced in the presence of denaturant lack an epitope that is expressed on polymer obtained from patient samples [18,21]. Given this diversity it is unsurprising that there are currently three main models for the terminal polymer that differ from one another in fundamental respects (Figure 1B). The RCL-to-β-sheet A linkage ‘loop-sheet’ model, supported by a considerable amount of biophysical data [2,6,8,10,22], has recently been joined by two models that are based on crystal structures of self-terminating oligomers: a circular dimer of an antithrombin mutant with an RCL-strand 5A β-hairpin domain swap [23], and a circular trimer of α_1_-antitrypsin formed in the presence of an engineered disulfide bond with a triple strand 1C–5B–6B domain swap [24]. The extent of the regions exchanged in these latter two structures has led to the proposal that the corresponding polymerization intermediates will involve substantial unfolding of some secondary structure elements.

While the main polymer models disagree in the nature of the inter-molecular linkage, they all predict an expanded 6-strand β-sheet A with RCL residues accommodated in the equivalent positions to that seen in the canonical cleaved conformation. This is consistent with the observation that α_1_-antitrypsin polymerization can be induced or blocked by incorporation of an exogenous peptide mimic of the RCL into β-sheet A [6,25,26]. The evidence therefore substantively implicates the RCL as a critical component of the mechanism of polymerization, while the extent of RCL insertion during intermediate (*I*_pol_) formation varies between the most parsimonious interpretations of the different models: partial or none in the case of the loop-sheet and the β-hairpin forms, and full insertion in the triple-strand model.

In order to assess the relative contribution of different RCL positions to *I*_pol_, we replaced the residues that are accommodated by β-sheet A in the cleaved and latent forms (P_14_, P_12_, P_10_,…, P_1′_) with aspartic acid [12] (Figure 1A). We determined the effect of these mutations on the rate of activation to the intermediate state by monitoring changes in tryptophan fluorescence, CD and binding to bis-ANS, and we report a novel FRET (Förster resonance energy transfer)-based polymerization assay.

Reconciliation of the results with features that are common to the three extant models of the polymer form reveals details of the pathway. An early structural transition, detectable by change in CD, precedes partial insertion of the P_14_P_12_ residues into β-sheet A. The interaction of the P_6_P_4_ residues with β-sheet A–whether in an inter- or intra-molecular fashion–occurs late in the polymerization pathway and presents an energetic barrier to polymerization.

The observation that polymerization in mammalian cells can be blocked by mutation of the P_6_P_4_ and P_14_P_12_ sites suggests that, *in vivo*, polymerization occurs from a state with intact strands 3A and 5A of β-sheets A, and strands 2B, 3B and 4B of β-sheets B. It is therefore concluded that most likely polymerization occurs from a near-native form rather than a substantially unfolded intermediate.

## EXPERIMENTAL

### Plasmid generation for the expression of recombinant α_1_-antitrypsin *in vitro* and in cell culture

The pQE-30 and pQE-81L plasmids (Qiagen) containing the ‘wild-type’ (M allele) α_1_-antitrypsin ORF with the C232S mutation (introducing N-terminal vector-derived amino acids MRGSHHHHHHT and MRGSHHHHHHTDPHASSVP, respectively) were used to express recombinant α_1_-antitrypsin [27]. The C232S substitution obviates the need for reducing agent in the assay buffer and was used as the control for *in vitro* experiments (AT_C232S_). This variant has been found to behave in an equivalent fashion to wild-type in the previous studies (for example [22]). Reactive loop mutants were prepared on the C232S background using aspartic acid-scanning mutagenesis [12] to generate T345D, A347D, G349D, M351D, L353D, A355D, P357D and S359D substitutions (corresponding with RCL positions P_14_, P_12_, P_10_, P_8_, P_6_, P_4_, P_2_ and P_1′_ in subsite notation [28]), yielding in combination the variants P_14_P_12_, P_10_P_8_, P_6_P_4_, P_2_P_1′_, P_6_ and P_4_. The pcDNA plasmid containing the Z (E342K) α_1_-antitrypsin allele was used as the basis for the mutants in cell culture experiments [29].

### Cell culture, transfection and lysis

COS-7 cells were purchased from the ATCC (American Type Tissue Culture Collection) and maintained in DMEM (Dulbecco's modified Eagle's medium) (Sigma) supplemented with 10% (v/v) FBS (Sigma). Two hours prior to transfection the medium was changed to Optimem (Gibco). The plasmid DNA containing wild-type, Z or mutants of α_1_-antitrypsin was transiently transfected into the COS-7 cells using lipofectamine LTX (Invitrogen). Cells were lysed after 24 h using 150 mM NaCl/50 mM Tris/0.1% (v/v) NP-40 with 25 mM Complete EDTA-free protease inhibitor (Roche Applied Science) and α_1_-antitrypsin polymers were detected by sandwich ELISA with the α_1_-antitrypsin polymer-specific 2C1 antibody as described previously [30].

### *Escherichia coli* expression and purification of recombinant α_1_-antitrypsin

Plasmids containing wild-type and mutated M α_1_-antitrypsin on a C232S background were transformed into SG13009/pREP4 cells and BL21 (DE3) cells (Novagen) for pQE-30 and pQE-81L-based constructs, respectively. Recombinant proteins were expressed and purified as described previously [30] before buffer exchange into 20 mM Tris, 100 mM NaCl, pH 7.4 and storage at −80°C. The resulting proteins were assessed by SDS- and non-denaturing PAGE, CD spectra, thermal stability and for their ability to inhibit bovine α-chymotrypsin. The different N-terminal extensions produced by the pQE-30 and pQE-81L expression systems generated α_1_-antitrypsin with the same thermal stability, secondary structure (as evaluated by CD), inhibitory kinetics and ability to form polymers as described previously [14,21,31].

### Thermal denaturation assay

The stability of α_1_-antitrypsin was investigated by thermal denaturation in the presence of a 5× concentration of SYPRO Orange dye solution (Life Technologies) in 50 mM Na_2_HPO_4_/NaH_2_PO_4_ buffer, pH 7.4, at a final protein concentration of 0.1 mg/ml and in a 20 μl volume [32]. Protein samples were heated from 25 to 95°C at a rate of 1°C min^−1^ in three separate experiments on an Applied Biosystems 7900HT quantitative real-time PCR instrument, and the fluorescence in the 625–630 nm bins recorded. The midpoint of denaturation (*T*_m_) was reported as the temperature at which the first derivative of fluorescence intensity against temperature reached a maximum.

### Assessment of inhibitory activity of α_1_-antitrypsin

Bovine α-chymotrypsin (Sigma) was titrated using 4-nitrophenyl acetate [33]. The SI (stoichiometry of inhibition) of wild-type and mutants of α_1_-antitrypsin was determined by incubation of the protein for 30 min at room temperature with 0.5 μM bovine α-chymotrypsin in 20 μl protease assay buffer (20 mM Tris, 100 mM NaCl, 0.1% (w/v) PEG 8000, 10 mM CaCl_2_, pH8.0). 180 μl of 200 μM N-succinyl-Ala-Ala-Pro-Phe-p-nitroanilide substrate (Sigma) was added and the rate of absorbance increase at 405 nm was recorded for 5 min using a ThermoMax plate reader (Molecular Devices). Linear regression was used to extrapolate the amount of inhibitor required to completely abrogate enzyme activity. The association rate constant of inhibitor with enzyme (*k*_ass_) was measured by reaction progress curves under pseudo-first-order conditions for 4 h at 25°C with a final concentration of 5–600 nM inhibitor, 200 μM substrate and 0.5 nM bovine α-chymotrypsin. Data analysis was as described previously [34].

### CD analysis

All far-UV spectra were obtained using a Jasco J-810 spectropolarimeter with a 0.2 mm pathlength cell. Samples were dialysed into 10 mM Na_2_HPO_4_/NaH_2_PO_4_, pH 7.4 and adjusted to 0.5 mg/ml prior to analysis. Scanning was performed between 260 and 180 nm at a rate of 50 nm/min, a 0.1 nm pitch and 1 s response time, with averaging of four spectra per experiment. A thermal shift assay to evaluate the effect of the ATTO-NTA (nitrilotriacetic acid) fluorescent probes (described below) was undertaken by monitoring the CD of the sample at 222 nm in a 2 mm pathlength cuvette, as the sample was heated from 5 to 95°C at a rate of 1°C min^−1^. The unimolecular activation assay used 0.1 mg ml^−1^ protein in 20 mM Na_2_HPO_4_/NaH_2_PO_4_, pH 7.4, heated at 55°C in a 10 mm pathlength cell while monitoring the output at 222 nm. Data were normalized as a proportion of the starting signal before fitting in Prism (GraphPad) to a two-phase exponential equation of the form

(1) $$I_{t}=1-Be^{-k_{1,\mathrm{cd}t}}-Ce^{-k_{2,\mathrm{cd}t}}$$

where *B* and *C* are pre-exponential factors, *k*_1,cd_ is the apparent rate of the early transition under the conditions of the assay and *k*_2,cd_ is the calculated rate of the late transition.

### Polymerization kinetics monitored by FRET

A microplate assay was developed in which the rate of polymer formation was determined by monitoring FRET between Ni-NTA (nickel-nitrilotriacetic acid)-conjugated ATTO-550 and ATTO-647N fluorescent probes (Sigma). These dyes are recruited from solution to interact non-covalently with the hexahistidine tag of the variants, and as such it is not possible to measure labelling efficiency; coupled with the heterogeneous nature of polymerization, no inference was therefore made as to the physical distance between fluorophores. α_1_-Antitrypsin was diluted to 0.1 mg/ml in polymerization buffer (10 mM Na_2_HPO_4_/NaH_2_PO_4_, 0.1 M sodium chloride, 0.1% (w/v) PEG8K, pH 7.4) containing a pool of 2–8 μM ATTO-550-Ni-NTA and 2–8 μM ATTO-647N-Ni-NTA probes. The ratio of fluorescence intensity of the donor and acceptor fluorophores was followed for 8–18 h at 50, 55 and 60°C. Change in fluorescence was monitored for 20 μl samples using an Applied Biosystems 7900HT quantitative PCR instrument (which uses a 488 nm laser to excite the donor) and the ratio of the emitted fluorescence in the 645–660 to 550–600 nm bins was monitored. FRET efficiency increased to a plateau value; in some cases this was followed by a gradual decrease interpreted as precipitation or secondary change in polymer characteristics. Data were normalized as a proportion of the initial FRET value; it was determined empirically that an overall increase of less than 5% corresponded with a lack of polymerization. As described in the Results section, it was found that best numerical stability was obtained for the fit, using Prism (Graphpad), of the integrated second-order rate equation for homogeneous reactants following truncation of the data below 25% of the total signal:

(2) $$\frac{F_{t}}{F_{0}}=A\left(1-\frac{1}{1+k_{\mathrm{app}.\mathrm{fr}}t}\right)+1$$

where *F_t_* denotes the ratio of acceptor to donor fluorescence (a measure of FRET efficiency) at time *t*, *F*_0_ is the starting FRET efficiency, *A* is a scaling factor and *k*_app,fr_ is the apparent rate constant under the conditions of the assay. For recording of full FRET spectra at room temperature, a SpectraMax M5 plate reader (Molecular Devices) was used, with excitation at 520 nm and monitoring emission between 550 and 725 nm with 4–10 nm intervals.

### Polymerization monitored by the change in intrinsic tryptophan fluorescence

A LS55B spectrofluorimeter (Perkin Elmer) fitted with a Peltier module was used to measure fluorescence of α_1_-antitrypsin at constant temperature in polymerization buffer. Samples were diluted 200–800-fold into pre-heated buffer to give an 800 μl final volume in a 4 mm×10 mm stirred quartz cuvette. Samples were excited at 290 nm and the emission was measured at 340 nm, with 3 nm widths, and the PMT voltage adjusted as appropriate. The resulting curves were fitted using a single exponential equation of the form:

(3) $$I_{t}=A+B\left(1-e^{-k_{\mathrm{app},\mathrm{fl}t}}\right)$$

in which *I_t_* is the fluorescence intensity at time *t*, *B* is the pre-exponential factor, *A* the baseline signal and *k*_app,fl_ the apparent rate of polymerization under the conditions of the assay.

### Monitoring the formation of an activated intermediate using bis-ANS

This experiment was conducted both in a microplate format using a SpectraMax M5 (Molecular Devices) plate reader and in a cuvette format using an LS55B spectrofluorimeter (Perkin Elmer). In both cases, polymerization buffer containing 10 μM bis-ANS and 0.1 μM fluorescein was pre-heated, samples diluted at least 20-fold, and measurement was initiated immediately. Final microplate well volume was 200 μl overlaid with 50 μl VaporLock and cuvette volume was 800 μl; excitation was at 390 nm and emission at 480 nm, with 2.5 nm/5 nm slit widths on the LS55B and fixed 9 nm/15 nm bandwidths on the M5 instrument. The fluorescein was used as a passive reference to correct for light path anomalies in the microplate assay and detected by exciting at 480 nm and reading the emission at 520 nm; the bis-ANS measurement was normalized against this value. Curves were fit to a single exponential equation.

## RESULTS AND DISCUSSION

### RCL mutants affect the accumulation of intracellular Z α_1_-antitrypsin polymers

While the precise role of the RCL in polymerization is contentious, its fundamental importance has been shown consistently in many studies [2,6,8,10,22–24]. The extant models of polymerization (Figure 1B) predict burial of at least some of the residues in the P_14_ to P_4_ region, and it has been found that oligomerization is blocked by peptides spanning P_7_-P_2_ [35,36] with the critical sites of interaction found to be P_6_ and P_4_ [37]. All models anticipate an interaction–inter- or intra-molecular–that is consistent with the positions of the inserted RCL residues in accordance with the canonical cleaved conformation (Figure 1A). We sought to probe the role of different RCL residues in the formation of polymers. Accordingly, four double mutants (P_14_P_12_, P_10_P_8_, P_6_P_4_ and P_2_P_1′_) and two single mutants (P_6_ and P_4_) were generated.

It is clear that the cellular machinery affects the polymerization process *in vivo*, as approximately 70% of synthesized Z α_1_-antitrypsin is degraded by endoplasmic reticulum-associated degradation [38]. In order to explore the effect of the mutants in this context, they were investigated using a COS-7 cell model that expresses the common severe Z mutant (E342K) of α_1_-antitrypsin. Cells were transiently transfected with pcDNA-based constructs to induce expression, and grown for 24 h before harvest; expressed protein levels were evaluated by sandwich ELISA.

The results revealed that while there was no significant difference in total intracellular α_1_-antitrypsin, the mutants exhibited marked variation in the accumulation of intracellular α_1_-antitrypsin polymers (Figure 1C), as detected by the 2C1 monoclonal antibody [21]. Substitutions at P_14_P_12_, P_6_P_4_ and P_4_ led to a marked decrease in polymer levels, but the reduction seen with the P_10_P_8_ variant did not reach statistical significance, and for the P_6_ and P_2_P_1′_ loop mutants, the intracellular polymer burden was equivalent to the Z α_1_-antitrypsin control. Notably, the P_6_P_4_ and P_4_ variants showed an equivalent repression of polymerization, demonstrating a lack of additive effect between the P_6_ and P_4_ positions. Overall, these data suggest that during polymerization of the Z variant within the ER, the P_10_–P_6_ positions represent a partition between two functionally distinct RCL regions, which potentially exert different effects on the polymerization mechanism.

### Mutations within the RCL exert a minimal effect on overall stability

**Figure 2:**
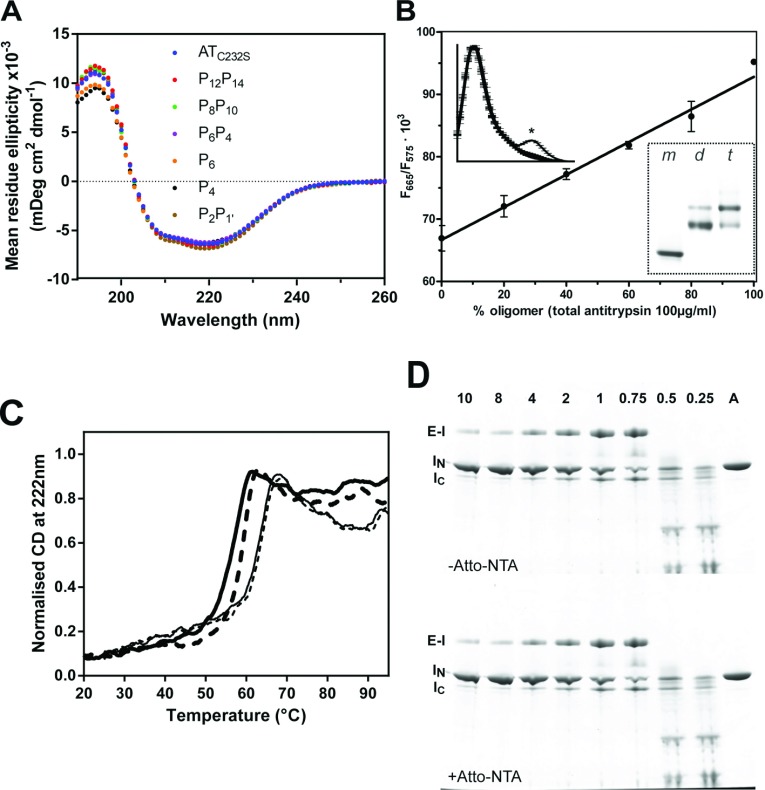
Characteristics of the mutants and heat-induced polymerization monitored by FRET (**A**) Assessment of the integrity of wild-type and reactive loop mutants by CD. CD spectra of the reactive loop mutants, at 0.5 mg/ml in 10 mM Na_2_HPO_4_/NaH_2_PO_4_ pH7.4, recorded between 260 and 190 nm, show a similar profile to that of the wild-type protein. The spectra are the average of at least four independent experiments. (**B**) Monomer and samples containing predominantly dimer or trimer were separated by gel filtration of polymerized material using a Superdex 200 column (inset gel). Different ratios of the oligomer fractions were mixed with monomer such that the total concentration of each sample was 0.1 mg/ml, and combined with 4 μM NTA-ATTO550 and 4 μM NTA-ATTO647N. The ratio of fluorescence at 665 nm to that at 575 nm upon excitation at 488 nm was recorded. The measurements shown are from two separate experiments, each using the two different oligomer preparations. The FRET signal varied inversely with the amount of monomer on non-denaturing PAGE. A linear relationship between oligomer concentration and FRET signal is in keeping with previous biophysical studies of α_1_-antitrypsin polymerization [14]. Inset fluorescence spectra, normalized for the emission maximum of ATTO550, show a distinct FRET peak (indicated by an asterisk ‘*’) for recombinant α_1_-antitrypsin heated at 60°C for 10 min in the presence of 4 μM NTA-ATTO550 and 4 μM NTA-ATTO647N dyes, but not for the unheated control or in the absence of either dye. (**C**) A 600 μl sample of recombinant α_1_-antitrypsin (thick lines) or plasma antitrypsin (thin lines) at 0.1 mg/ml in 10 mM Na_2_HPO_4_/NaH_2_PO_4_, 100 mM NaCl in the presence (unbroken lines) or absence (broken lines) of 4 μM NTA-ATTO550/4 μM NTA-ATTO647N was heated from 20 to 95°C at a rate of 1°C min^−1^ in a Jasco-J810 spectropolarimeter and the ellipticity at 222 nm measured across a 2 mm pathlength. Values were scaled to occur between 0 and 1.0; curves shown are the average of two experiments and error bars indicate the difference between the duplicate measurements. The midpoint of denaturation of recombinant α_1_-antitrypsin in these buffer conditions is 59.6±0.1°C in the absence of the reporter dyes and 58.0±0.4°C in the presence of the dyes, and the respective midpoints for the plasma protein are 64.0±0.1 and 63.8±0.1°C. (**D**) 2 μg α_1_-antitrypsin (lane A) was combined with different molar ratios of bovine α-chymotrypsin (indicated by numbers at the top of the gel) in chymotrypsin assay buffer for 15 min at room temperature. Samples were mixed with SDS loading buffer without boiling and separated on an SDS/PAGE(4–12% gel) bis-Tris PAGE, and visualized by Coomassie Brilliant Blue. The position of the α_1_-antitrypsin–bovine α-chymotrypsin complex and α_1_-antitrypsin in the native and cleaved forms are indicated by the *E*–*I*, *I*_N_ and *I*_C_ labels, respectively.

In order to explore in detail the effect of the mutations on the polymerization of α_1_-antitrypsin, the variants were produced using an *E. coli* expression system. The control protein used in all *in vitro* experiments was a C232S point mutant of the M allele of antitrypsin (AT_C232S_) which has been found to behave identically to wild-type protein [22]. CD in the far-UV range was used to determine whether the mutants had an altered secondary structure. The loop mutants were all found to share a similar far UV CD profile to AT_C232S_ (Figure 2A), and had melting temperatures in a SYPRO Orange stability assay that were no greater than 2.0°C above that of the control protein (Table 1). In contrast, almost no inhibitory activity against chymotrypsin was observed for any of the mutants, with the exception of P_10_P_8_, which displayed approximately 20% of wild-type activity (Table 1). This is consistent with the mutated sites affecting the ability to insert into β-sheet A while not affecting overall structure in the native conformation and having a minimal effect on global native state stability.

**Table 1:** Biophysical and biochemical characteristics of recombinant wild-type and RCL mutants of α_1_-antitrypsin Variants were assessed for their midpoint of denaturation, SI and association rate constant (*k*_ass_) against bovine α-chymotrypsin. The results are the mean of at least three independent experiments. n.i., non-inhibitory (less than 1% residual inhibitory activity).

Variant	*T*_m_ (°C)*	SI†	*k*_ass_ (M^−1^ s^−1^)	*k*_ass_•SI
AT_C232S_	55.0	1.1±0.02	1.5±0.2×10^6^	1.5×10^6^
P_2_P_1′_	55.0	100±9	–	–
P_6_	54.5	n.i.	–	–
P_4_	55.0	n.i.	–	–
P_6_P_4_	55.0	89±7	–	–
P_10_P_8_	56.0	4.56±0.32	1.2±0.2×10^5^	5.7×10^5^
P_14_P_12_	57.0	n.i.	–	–

*All standard errors were less than precision of the technique on the instrument (±0.5°C).

^†^Standard errors were calculated by regression of a transformed linear equation with the intercept at the abscissa as a parameter.

### Development of a FRET-based assay to follow oligomerization

Spectroscopically, serpin polymerization is typically followed using approaches that monitor coincident changes in the intrinsic properties of the molecule. We sought to use a technique that directly reports increases in physical proximity during oligomerization. For this purpose, we developed a FRET-based assay by exploiting the presence of a hexa-histidine affinity tag on the recombinant proteins. Ni-NTA-conjugated ATTO-550 and 647N are commercially available dyes that are able to interact non-covalently with these affinity tags. In the presence of pre-polymerized α_1_-antitrypsin, NTA-ATTO-550 was found to excite NTA-ATTO-647N via FRET (Figure 2B, inset top left), with the magnitude of the signal proportional to the amount of oligomer present (Figure 2B, graph). The presence of the dyes led to a 1.6°C decrease in thermal stability as determined using a CD-based thermal shift assay (Figure 2C), but had no effect on the ability to form SDS-stable complexes with bovine α-chymotrypsin (Figure 2D). It has previously been noted that the hexa-histidine tag increases stability of the recombinant protein [14], and this probably represents a partial negation of this effect, as the presence of the dye had no effect on the thermal stability of non-tagged plasma α_1_-antitrypsin (Figure 2C).

**Figure 3:**
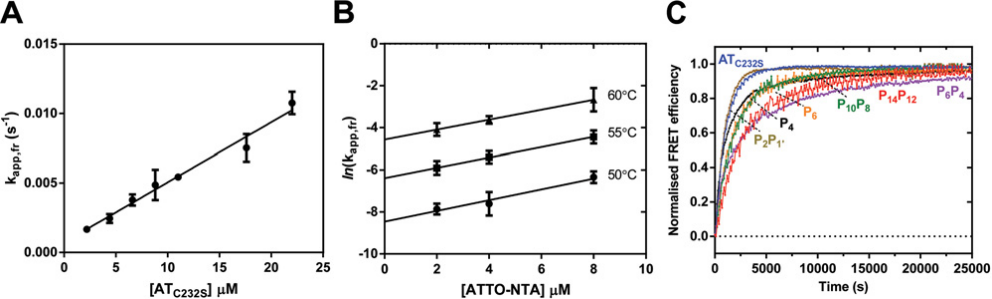
Characterization of the FRET assay (**A**) The FRET-based polymerization assay was conducted using different concentrations of AT_C232S_ at 55°C in the presence of 2 μM dyes, and the resulting rate constants, *k*_app,fr_, were plotted. (**B**) The rate of polymerization reported by the FRET assay varied as a function of dye concentration. Linear regression of the natural logarithm of the apparent rate constant, *k*_app,fr_, permitted extrapolation of the rate in a dye-free solution, shown for the control (AT_C232S_) protein at 50, 55 and 60°C. Each data point is the result of at least three independent experiments and error bars show the standard error of the mean. (**C**) Polymerization progress curves for recombinant control α_1_-antitrypsin and loop variants at 55°C were corrected for the effect of the NTA-ATTO dyes on apparent rate, as described in the text, and averaged. Samples were prepared at a concentration of 0.1 mg/ml in polymerization buffer with 2–8 μM NTA-ATTO550 and NTA-ATTO647N. Fluorescence was monitored continuously on an ABI 7900HT instrument upon excitation with the 488 nm laser and the FRET efficiency calculated; the normalized values are shown as the fraction of polymerized material present in the sample.

In conjunction with a laser-excited real-time thermal cycler, it was possible to monitor the formation of polymer of multiple low-volume (20 μl) samples concurrently in a microplate assay (Figure 3A). Non-linear regression analysis was performed using differential equations describing several alternative possible reaction schemes with COPASI [39]. The simplest kinetic reaction scheme that minimized the RMS deviation from the data was:


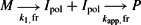


where *k*_1,fr_ is a first-order activation rate constant, *k*_app,fr_ is the apparent second-order association rate constant, M is a non-activated monomer, *I*_pol_ a polymerization intermediate and *P* the species reported by FRET. Exploration of alternative pathways with greater complexity, including reversible activation and mixed intermediate species, failed to yield improved fits; similarly, removal of the initial unimolecular step resulted in a much poorer correspondence with the data.

It was ultimately found that a value for *k*_app,fr_ could be obtained with optimal numerical stability by fitting the integrated second-order rate equation (as detailed in the Materials and Methods section). However, it was not possible to derive robust values for *k*_1,fr_ using this method, in which the presence of *I*_pol_ could only be inferred from the lack of conformity of an initial part of the curve to the second-order rate model. As equation (2) implicitly represents the concentration of polymer at a given time point as a proportion of a final steady-state value, points could be excluded below a nominal threshold value to ensure that *k*_app,fr_ was determined from a region subject only to second-order time dependence. It was found that a cutoff of 25% of the plateau FRET signal maximized the number of data points considered while effectively excluding the region of the curve subject to the first-order conversion of *M* to *I*_pol_.

The resulting second-order rates were proportional to α_1_-antitrypsin concentration (Figure 3A), confirming that the assay was reporting a concentration-dependent bi-molecular step. The slope of the regression corresponds with a rate of polymerization of 4.3±0.3×10^2^ M^−1^ s^−1^. The linearity of this relationship, in the context of the reaction scheme shown above, indicates that the activation step was not rate-limiting.

When experiments were performed in the presence of different concentrations of ATTO-NTA, a linear relationship was also found between the natural logarithm of *k*_app,fr_ and the dye concentration used (Figure 3B). This enabled the derivation of a theoretical rate in the absence of dye. The resulting corrected rates were in excellent agreement with other measures of polymerization, as detailed below.

### Mutations at P_6_P_4_ increase the activation energy of polymerization

Progress curves were generated using the FRET-based assay for each α_1_-antitrypsin mutant at 50, 55 and 60°C at a fixed concentration of 0.1 mg/ml (Figure 3C), and the dye-corrected apparent rate constant, *k*_app,fr_, was determined under each condition (Table 2). At least three independent experiments were performed at each temperature at three concentrations of dye. The mutants displayed a range of behaviours, demonstrating differential effects of the mutations at different sites in the RCL. Only P_2_P_1′_ behaved in a wild-type fashion. At 50°C, the rate of polymerization followed the order P_6_P_4_<P_6_<P_14_P_12_<P_10_P_8_<P_4_<AT_C232S_/P_2_P_1_, from slowest to fastest. At 55°C, P_6_P_4_ and P_14_P_12_ were found to be the most resistant to polymer formation. At 60°C, the barrier to polymerization was sufficiently reduced such that the variants in the P_6-_P_1′_ range demonstrated similar rates to the control; however, the P_14_P_12_ and P_10_P_8_ variants remained slower. It is interesting to note that the behaviours of the P_6_ and P_4_ variants deviate in this assay from that observed in cells in the presence of the Z mutation (Figure 1). As oligomers in both contexts are recognized by the 2C1 anti-pathogenic polymer antibody, it is unlikely that this is the product of fundamentally different mechanisms of polymerization; however it does indicate there are differences in the molecular details. This may reflect the difference between a specific destabilization of the top of β-sheet A by the Z point mutation [6,7,17] and a more general destabilization of the β-sheet A under thermal stress.

**Table 2:** Effect of mutations on polymerization The apparent rate of polymerization, determined using the FRET-based assay, is shown for each of the α_1_-antitrypsin loop mutants, at 0.1 mg ml^−1^. The aggregate number of independent observations for a variant at each temperature is shown, and standard errors are reported. The apparent energy of activation was calculated by application of Arrhenius’ law.

	*k*_app,fr_ (s^−1^)						
Variant	50°C	*n*	55°C	*n*	60°C	*n*	*E*_act_ (kJ mol^−1^)
AT_C232S_	2.1±0.30×10^−4^	31	1.6±0.17×10^−3^	32	1.0±0.11×10^−2^	41	3.6±0.12×10^2^
P_14_P_12_	0.58±0.074×10^−4^	18	0.43±0.18×10^−3^	17	2.4±0.020×10^−2^	18	3.3±0.32×10^2^
P_10_P_8_	0.72±0.15×10^−4^	20	0.66±0.21×10^−3^	18	0.28±0.026×10^−2^	18	3.3±0.29×10^2^
P_6_P_4_	0.098±0.075×10^−4^	15	0.44±0.10×10^−3^	29	0.55±0.12×10^−2^	21	5.5±0.53×10^2^
P_6_	0.37±0.071×10^−4^	16	0.70±0.14×10^−3^	20	1.0±0.37×10^−2^	17	5.0±0.34×10^2^
P_4_	1.1±0.47×10^−4^	13	1.1±0.28×10^−3^	32	7.8±0.27×10^−2^	17	3.8±0.47×10^2^
P_2_P_1′_	2.2±0.79×10^−4^	13	1.7±0.15×10^−3^	22	1.2±0.21×10^−2^	18	3.6±0.26×10^2^

**Figure 4:**
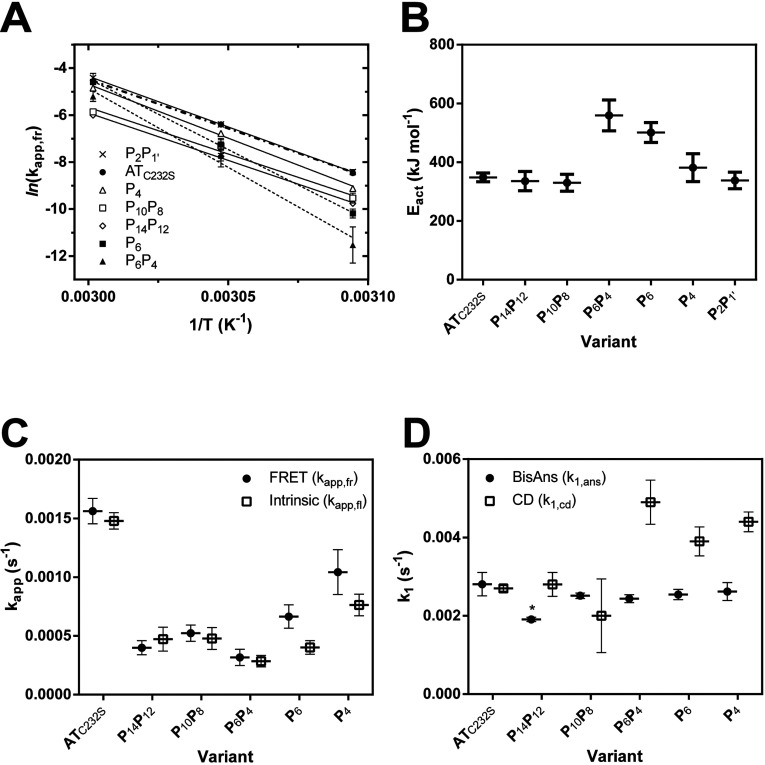
Apparent rate constants calculated for activation and polymerization of control α_1_-antitrypsin and reactive loop mutants (**A**) The inverse relationship between the natural logarithm of the apparent rate of polymerization, *k*_app,fr_ (calculated for each variant as shown in Figure 2D), and temperature was used to determine the energy of activation of the reaction, *E*_act_. Each data point is from least ten independent experiments. (**B**) The calculated energy of activation, *E*_act_, for the heat-induced polymerization of each variant is shown. (**C**) The change in intrinsic tryptophan fluorescence was monitored for the variants at 55°C using an LS55B instrument with a stirred cuvette and excitation at 280 nm and emission at 340 nm (open squares). Protein concentration was 0.1 mg/ml in 800 μl polymerization buffer. Values shown (*k*_app,fl_) are from three to five independent experiments and are the result of fitting a single exponential equation to the data [14]. The combined results of the FRET assay at this temperature are shown for comparison (*k*_app,fr_) interpolated from the linear regressions in panel (A) (closed circles). (**D**) The change in fluorescence in the presence of 10 μM bis-ANS was followed at 55°C in a cuvette or plate reader format, and fitted to a single exponential equation (closed circles). The resulting rates (*k*_1,ans_) were calculated from seven independent experiments. Alongside these values are the rates of change of circular dichroic ellipticity at 222 nm (*k*_1,cd_), calculated from two to three independent experiments for each point (open squares). An asterisk ‘*’ indicates a significant decrease (*P*=0.035) with respect to the control as determined by one-way ANOVA using the Bonferroni multiple test correction.

In accordance with Arrhenius’ law, a linear relationship was found between the natural logarithm of the rates and the inverse of the experimental temperature (Figure 4A). From the slopes of these regressions, it was possible to calculate the apparent energy of activation (*E*_act_) for the polymerization reaction. The AT_C232S_ control variant was determined to have an activation energy of polymerization of ~360 kJ mol^−1^ (Table 2). This analysis revealed a striking behaviour for the P_6_ and P_6_P_4_ mutants: steeper curves with respect to the other variants were consistent with an additional barrier to activation of 140–190 kJ mol^−1^. Notably, despite the considerable resistance of the P_10_P_8_ and P_14_P_12_ α_1_-antitrypsin variants to polymerization, these mutants had comparable activation energies to the AT_C232S_ control (Figure 4B and Table 2), indicating their effects are manifested during the unimolecular activation phase rather than the multimolecular association phase.

### Congruence between FRET and intrinsic fluorescence-based assays of polymerization

As polymerization is the consequence of distinct activation and self-association processes, we sought to determine whether the effects of the mutations could be explained by differences in intermediate formation. It has been observed previously that intermediate formation can be followed spectroscopically by CD, ANS or bis-ANS binding, and by two-phase analysis of change in intrinsic tryptophan fluorescence [14,40].

Unimolecular activation of plasma-derived α_1_-antitrypsin is associated with an increase in fluorescence which is of intermediate magnitude to that seen with subsequent polymerization [14]. This indicates that distinct changes in the tryptophan environment occur during each phase. For all of the variants, at 55°C tryptophan fluorescence was found to follow only a single exponential function. This lack of a detectable second ‘fast’ phase for the recombinant protein, when compared with glycosylated plasma-derived α_1_-antitrypsin, has been described previously [14]. Thus, the fluorescence signal is dominated by the spectroscopic changes that occur during the multimolecular association phase. It is noteworthy that the fitted rates (*k*_app,fl_) were in close agreement with those obtained using the FRET approach (Figure 4C), which is a direct measure of the increase in physical proximity associated with oligomerization. In addition to providing independent support for the utility of the FRET method, this result further affirms that intrinsic fluorescence reports only the self-association phase of the pathway for recombinant α_1_-antitrypsin.

### bis-ANS reports a decreased rate of activation for P_14_P_12_ α_1_-antitrypsin

At 55°C, the bis-ANS dye was found to report a phase with significantly faster rates (*k*_1,ans_) for all proteins than that reported by the FRET and intrinsic fluorescence assays (Figure 4D, circles). This is consistent with its ability to detect an activation step that precedes polymer formation [40]. Interestingly, however, only P_14_P_12_ showed a significant decrease in the rate of activation with respect to the AT_C232S_ control (one-way ANOVA, *P*<0.05). These residues are known in other serpins to partially insert into the top of β-sheet A, and are the first to insert during an inhibitory interaction. Combined with the observation that at non-polymerizing temperatures, bis-ANS is able to interact with the Z variant of α_1_-antitrypsin but not wild-type [17], it is probably that this difference in rate reflects an early activation step involving the opening of β-sheet A and initial insertion of these residues of the RCL.

### Differential effects on the rate of circular dichroic change during polymerization

The unimolecular phase of polymerization can be followed using CD spectroscopy at 222 nm, which reports changes in secondary structure content [14]. As the bis-ANS data did not account for differences in the rate of polymerization, and since the use of an ANS-based dye may perturb structural equilibria [19], we used this technique to explore other potential effects on the activation to *I*_pol_.

**Figure 5:**
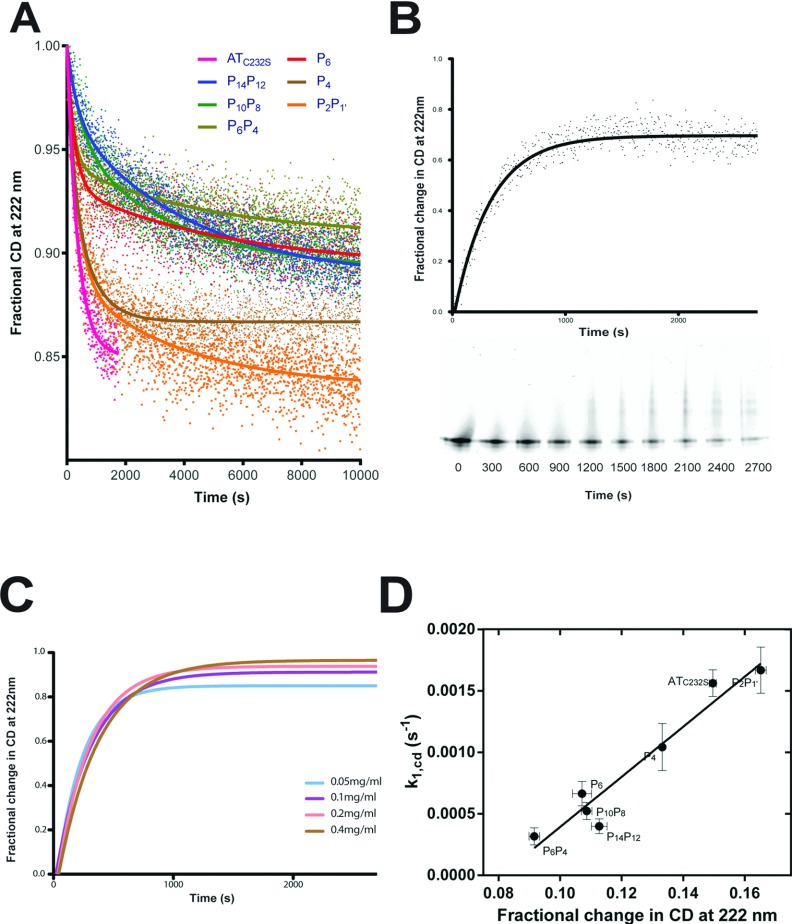
Intermediate formation monitored by CD (**A**) Change in secondary structure content was monitored at 222 nm in a 2 mm pathlength cuvette containing 20 mM Na_2_HPO_4_/NaH_2_PO_4_ pH 7.4 pre-warmed to 55°C. The progress curves shown are the aggregate of three replicate experiments, normalized to the starting CD signal, and fit using a two-phase exponential equation. (**B**) The normalized change in ellipticity for the AT_C232S_ control at 55°C is shown. Periodically, an aliquot containing 2 μg of material was removed and snap frozen, before separation on a 4–12% (w/v) acrylamide non-denaturing gel; positions in the figure are relative to the time at which they were removed. (**C**) The rate of change of the CD signal at 55°C at different concentrations of AT_C232S_ is shown. (**D**) A trend is evident between the magnitude of the CD signal change and the rate of polymerization of the variants.

A pronounced initial decrease in ellipticity for all proteins was observed within approximately 1200 s (Figure 5A), during which time non-denaturing gel analysis revealed minimal polymer formation (Figure 5B). Varying serpin concentration failed to have a significant effect on the shape of the curve (Figure 5C) over this time frame, suggesting CD indeed reports a pre-oligomerization activation step. The CD data were expressed as a function of starting ellipticity, and fit to a two-phase exponential decay equation. Surprisingly, the P_10_P_8_ and P_14_P_12_ mutants were not significantly different in the early ‘fast’ phase from the AT_C232S_ control, and the P_6_, P_4_ and P_6_P_4_ variants exhibited an increased rate despite their repression of polymerization (*k*_1,cd_ in Table 3). This conflicting behaviour indicates that the underlying structural change represented by the increase in CD is not a rate-limiting step in the polymerization of these variants.

**Table 3:** Rate and magnitude of the structural change reported by CD at 55°C The plateau value reflects the magnitude of the change from the starting CD ellipticity measurement. Parameters were calculated from fitting a two-phase exponential equation to the combined data from at least three independent experiments. Standard errors from the fit are reported.

Variant	*k*_1,cd_ (s^−1^)	*k*_2,cd_ (s^−1^)	Plateau
AT_C232S_	2.7±0.07×10^−3^	–	0.85±0.0011
P_14_P_12_	2.8±0.31×10^−3^	2.4±0.26×10^−4^	0.89±0.0031
P_10_P_8_	2.0±0.94×10^−3^	2.8±0.34×10^−4^	0.87±0.00029
P_6_P_4_	4.9±0.56×10^−3^	2.1±0.30×10^−4^	0.89±0.0025
P_6_	3.9±0.37×10^−3^	1.8±0.36×10^−4^	0.91±0.0017
P_4_	4.4±0.25×10^−3^	14±1.7×10^−4^	0.89±0.0017
P_2_P_1′_	2.9±0.14×10^−3^	2.6±0.32×10^−4^	0.83±0.0017

It has been noted previously that CD of plasma α_1_-antitrypsin reports a single transition consistent with activation and not polymerization [14]. The behaviour of the control variant is consistent with this. While the CD progress curves of the mutants demonstrated a measurable second phase, the significance of this was not clear, as the rate (*k*_2,cd_) did not appear to correlate with other measures of polymerization or activation (Table 3). This transition may therefore represent a secondary structural change not directly associated with the polymerization mechanism.

The amplitudes of the curves themselves reflect differing overall degrees of secondary structural change over the course of the experiment. It was found that the magnitude of this change showed a strong linear trend against the rate of polymerization of the variants (Table 3 and Figure 5D). At a molecular level, this could be the result of α_1_-antitrypsin molecules adopting two distinct structural states that contribute differentially to the CD of the sample. It is unlikely that these two states represent sequential intermediates on a single reaction path, as the progress curves do not converge to a common CD value; this lack of convergence also indicates that the different contributions to the CD signal persist during polymer formation. When expressed as the fractional deviation from the starting CD value, extrapolation of the linear regression to *k*_1,cd_=0 s^−1^ yielded an intercept of 0.0807, indicating that one of the components had lost about 8% of the measured secondary structure signal at 222 nm with respect to the monomer.

Although this analysis was not possible for the second component, it could be inferred from the linear relationship that the second component is required for polymerization to occur, that is, the first component is unable to self-associate. Polymerization by association between ‘donor’ and ‘acceptor’ molecules could be one explanation for these observations. Differences in population of these two states would accordingly influence the apparent rate of inter-molecular association, and explain why, in the absence of the increased energy of activation observed with the P_6_P_4_ and P_6_ variants, the P_14_P_12_, P_10_P_8_ and P_4_ variants result in a reduced rate of polymerization.

### Conclusions: the dichotomous behaviour of the RCL mutants

Mutations in distinct regions of the RCL of the Z variant of α_1_-antitrypsin–at P_14_P_12_ and P_4_–are able to significantly affect accumulation of polymers in cells. *In vitro*, the P_14_P_12_ mutant decreases the rate of polymerization, in part by interfering with an initial insertion of the RCL at an early stage in the pathway. This effect is consistent with the three extant models of polymerization (Figure 1B). In the context of the RCL-exchange models, pre-insertion of the P_12_ and P_14_ residues coincides with opening of β-sheet A, which in the case of the loop-sheet polymer, would ultimately accommodate the loop of a second molecule. In the β-hairpin model, this step foreshadows the destabilization and loss of strand 5A. When considered, on the other hand, through the prism of the C-terminal polymer, this insertion step precedes a cascading RCL as the molecule transitions to a latent-like conformation.

The effectiveness of this mutant as a polymerization blocker in cells has an important consequence for the polymerization pathway *in vivo*. The P_14_ and P_12_ positions, in the partially inserted form, are situated in close proximity to the strands 4B and 5B that are displaced in the C-terminal model, and their mainchain atoms form hydrogen bonds with adjacent β-strands 3A and 5A. This strongly suggests that, regardless of the polymer model considered, the polymerizing species is in a near-native state with respect to the ‘breach’ region at the top of β-sheet A.

In contrast, P_6_P_4_ does not decrease the rate of early changes reported by bis-ANS or CD, but increases the activation energy required to transit between the intermediate ensemble these methods report, and the terminal self-associated form. As this increase is reflected in the intermolecular rate constant, this is most likely the consequence of interference with an intermolecular interaction. It is nevertheless possible that the mutant interferes with the progression to a late-stage intermediate form; however it is noteworthy that the P_14_P_12_ variant shows no effects on the activation energy despite directly affecting the bis-ANS-binding intermediate state, arguing against significant influence of the first-order step on the calculation of the second-order rate constant.

In the case of the C-terminal polymer, self-insertion of P_6_P_4_ would represent a kinetic barrier in the transition to the latent-like activated species. At this stage, strand 1C and possibly the C-terminus will need be disengaged, and the situation of the incoming P_6_P_4_ residues in the bottom half of the A-sheet beneath the F-helix and hF-s3A loop may amplify the energetic cost of the mutations (Figure 1A, right panel). Loop-exchange models suggest that the requirement to partly displace this helix would enhance the ability of the mutations to interfere with the *intermolecular* association. A common underlying interpretation is that the structural character of this region, which includes the F-helix and hF-s3A loop, provides an energetic barrier at the late stage of polymer formation. The efficacy of this mutant in cells therefore supports a polymerogenic species with an intact F-helix and underlying β-sheet A.

It is noteworthy that the P_10_P_8_ mutant has only an intermediate effect on polymerization. These residues are expected to be positioned near the ‘shutter’ region that regulates opening of β-sheet A during insertion, and are situated in close proximity to the C-terminal portion of the F-helix that has been found to undergo remodelling during polymerization [41]. In light of the observation that this mutant retains some inhibitory activity, it may be that once sheet opening has been initiated, these positions have a limited ability to prevent self-insertion (C-terminal model) or that they play a minor role in any possible inter-molecular interactions (loop-exchange models). Indeed, when the latter models are considered, it appears that in the presence of the Z mutation that the bridging ‘linker’ region is more extensive than in heat-generated polymers and includes P_6_ as well as the P_10_P_8_ residues.

In conclusion, these data support a polymerization reaction scheme in which a structural transition precedes the opening of β-sheet A, followed by an obligate partial insertion of the P_14_P_12_ residues, and ultimately a kinetic barrier involving the insertion of residues P_6_P_4_ into β-sheet A. CD provides evidence for a direct link between intermediate structure and polymerization rate. Finally, in cells, polymerization most likely proceeds from a near-native state.
