# Reactive centre loop mutants of α-1-antitrypsin reveal position-specific effects on intermediate formation along the polymerization pathway

**Published:** 2013-08-23

**Authors:** Imran Haq, James A. Irving, Sarah V. Faull, Jennifer A. Dickens, Adriana Ordóñez, Didier Belorgey, Bibek Gooptu, David A. Lomas

Volume 33, issue 3, e00046

In the published version of this paper, some errors were introduced by the publisher.

Equations 1 and 3 were incorrect. The correct equations are below.

Equation 1:

$$I_{t}=A-Be^{-k_{1,\mathrm{cd}}t}-Ce^{-k_{2,\mathrm{cd}}t}$$

Equation 3:

$$I_{t}=A+B\left(1-e^{-k_{\mathrm{app},\mathrm{fl}}t}\right)$$

Some values in Table 2 were incorrect. The correct version of Table 2 is below.

**Table:** 

	*k*_app,fr_ (s^−1^)						
Variant	50°C	*n*	55°C	*n*	60°C	*n*	*E*_act_ (kJ mol^−1^)
AT_C232S_	2.1±0.30×10^−4^	31	1.6±0.17×10^−3^	32	1.0±0.11×10^−2^	41	3.6±0.12×10^2^
P_14_P_12_	0.58±0.074×10^−4^	18	0.43±0.18×10^−3^	17	0.24±0.020×10^−2^	18	3.3±0.32×10^2^
P_10_P_8_	0.72±0.15×10^−4^	20	0.66±0.21×10^−3^	18	0.28±0.026×10^−2^	18	3.3±0.29×10^2^
P_6_P_4_	0.098±0.075×10^−4^	15	0.44±0.10×10^−3^	29	0.55±0.12×10^−2^	21	5.5±0.53×10^2^
P_6_	0.37±0.071×10^−4^	16	0.70±0.14×10^−3^	20	1.0±0.37×10^−2^	17	5.0±0.34×10^2^
P_4_	1.1±0.47×10^−4^	13	1.1±0.28×10^−3^	32	0.78±0.27×10^−2^	17	3.8±0.47×10^2^
P_2_P_1′_	2.2±0.79×10^−4^	13	1.7±0.15×10^−3^	22	1.2±0.21×10^−2^	18	3.6±0.26×10^2^

